# Annual and Weekly Incidence Rates of Influenza and Pediatric Diseases Estimated from Infectious Disease Surveillance Data in Japan, 2002-2005

**DOI:** 10.2188/jea.17.S32

**Published:** 2008-01-30

**Authors:** Miyuki Kawado, Shuji Hashimoto, Yoshitaka Murakami, Michiko Izumida, Akiko Ohta, Yuki Tada, Mika Shigematsu, Yoshinori Yasui, Kiyosu Taniguchi, Masaki Nagai

**Affiliations:** 1 Department of Hygiene, Fujita Health University School of Medicine.; 2 Department of Health Science, Shiga University of Medical Science.; 3 Department of Public Health, Saitama Medical University Faculty of Medicine.; 4 Infectious Disease Surveillance Center, National Institute of Infectious Diseases.

**Keywords:** Sentinel Surveillance, Incidence, Influenza, Human

## Abstract

**BACKGROUND:**

The method for estimating incidence of infectious diseases from sentinel surveillance data has been proposed. In Japan, although the annual incidence rates of influenza and pediatric diseases estimated using the method were reported, their weekly incidence rates have not.

**METHODS:**

The weekly sex- and age-specific numbers of cases in the sentinel medical institutions in the National Epidemiological Surveillance of Infectious Diseases in Japan in 2002-2005 were used. Annual and weekly incidence rates of influenza and 12 pediatric diseases were estimated by the above-mentioned method, under the assumption that sentinels are randomly selected from all medical institutions.

**RESULTS:**

The annual incidence rate of influenza in 2002-2005 was 57.7-142.6 per 1,000 population. The highest weekly incidence rate was 7.4 at week 8 in 2002, 14.9 at week 4 in 2003, 14.1 at week 5 in 2004, and 21.2 at week 9 in 2005. The annual incidence rate per 1,000 population of 0-14 years old in 2002-2005 was less than 5.0 for pertussis, rubella and measles, 293.2-320.8 for infectious gastroenteritis, and 5.3-89.6 for 8 other diseases. The highest weekly incidence rate was less than 1.0 for exanthem subitum, and was more than 5.0 for infectious gastroenteritis, hand-foot-mouth disease and herpangina.

**CONCLUSION:**

We estimated annual and weekly incidence rates of influenza and pediatric diseases in Japan in 2002-2005, and described their temporal variation.

In infectious diseases with large seasonal variation, such as influenza or measles, the annual and monthly or weekly incidence rate is essential for public health practice. The magnitude and temporal accumulation of such disease epidemics in a population, which would be important for planning control of epidemics, were observed in annual and monthly/weekly incidence rates. National infectious disease surveillance has been established in many countries.^1^^-^^8^ The incidence rate of a targeted disease is obtained directly from the surveillance data completely covering its occurrence, but it is not calculated directly from the data of sentinel surveillance. A method for estimating incidence rate of infectious disease from sentinel surveillance data has been proposed.^9^^-^^12^

In Japan, sentinel surveillance for influenza and pediatric diseases is conducted as a part of the National Epidemiological Surveillance of Infectious Diseases (NESID).^13^^-^^15^ The annual incidence rates of these diseases in 2002-2004 estimated from the sentinel surveillance data using the proposed method were reported,^16^ but the weekly incidence rates are not yet clear.

In the present study, we estimated annual and weekly incidence rates of influenza and pediatric diseases from the NESID data in Japan in 2002-2005, using the proposed method.

## METHODS

### Surveillance of Infectious Diseases in Japan

General outline of the NESID in Japan has been described elsewhere.^13^^-^^15^ Since 1999, the NESID has targeted influenza and 12 pediatric diseases (shown in Table 3) for sentinel surveillance. Local governments (prefectures) select sentinel medical institutions for influenza and pediatric diseases according to the NESID guidelines. The numbers of sentinels in the areas covered by pu lic health centers are approximately proportional to their population sizes. Each sentinel reports to a local public health center weekly. The report includes the sex- and age-specific numbers of cases newly diagnosed during a given week.

**Table 3.** Estimated incidence rates of pediatric diseases in population aged 0-14 years, Japan, 2002-2005.

Disease	Year	Estimated incidence	Incidence rate (per 1,000 population aged 0-14 years)	

			Estimate	95% confidence interval
Pharygoconjunctival fever	2002	99,000	5.5	4.6 - 6.5
	2003	256,000	14.3	12.3 - 16.4
	2004	383,000	21.4	18.6 - 24.1
	2005	397,000	22.2	18.4 - 26.0

Group A streptococcal pharyngitis	2002	929,000	51.9	47.2 - 56.5
	2003	995,000	55.6	51.0 - 60.1
	2004	1,244,000	69.5	62.3 - 76.6
	2005	1,192,000	66.6	60.5 - 72.6

Infectious gastroenteritis	2002	5,249,000	293.1	273.3 - 313.0
	2003	5,405,000	301.9	280.1 - 323.6
	2004	5,744,000	320.8	296.8 - 344.8
	2005	5,639,000	314.9	293.7 - 336.2

Chickenpox	2002	1,605,000	89.6	85.1 - 94.2
	2003	1,481,000	82.7	78.1 - 87.3
	2004	1,474,000	82.3	77.9 - 86.7
	2005	1,542,000	86.1	81.8 - 90.4

Hand-foot-mouth disease	2002	570,000	31.8	29.9 - 33.8
	2003	1,027,000	57.4	54.2 - 60.5
	2004	527,000	29.4	27.3 - 31.6
	2005	657,000	36.7	34.1 - 39.3

Erythema infectiosum	2002	369,000	20.6	19.2 - 22.1
	2003	205,000	11.4	10.6 - 12.3
	2004	308,000	17.2	15.8 - 18.5
	2005	272,000	15.2	13.9 - 16.6

Exanthem subitum	2002	687,000	38.4	36.0 - 40.7
	2003	682,000	38.1	35.6 - 40.5
	2004	685,000	38.3	35.4 - 41.1
	2005	689,000	38.5	36.0 - 41.0

Pertussis	2002	9,000	0.5	0.4 - 0.6
	2003	8,000	0.4	0.4 - 0.6
	2004	12,000	0.7	0.6 - 0.8
	2005	9,000	0.5	0.4 - 0.6

Rubella	2002	18,000	1.0	0.8 - 1.3
	2003	17,000	0.9	0.7 - 1.1
	2004	30,000	1.7	1.2 - 2.2
	2005	10,000	0.6	0.4 - 0.7

Herpangina	2002	695,000	38.8	36.0 - 41.7
	2003	912,000	50.9	47.3 - 54.5
	2004	659,000	36.8	33.7 - 39.9
	2005	926,000	51.7	47.8 - 55.6

Measles	2002	72,000	4.0	3.6 - 4.4
	2003	48,000	2.7	2.3 - 3.0
	2004	10,000	0.6	0.4 - 0.7
	2005	6,000	0.3	0.3 - 0.4

Mumps	2002	1,045,000	58.4	54.9 - 61.8
	2003	492,000	27.5	25.6 - 29.4
	2004	789,000	44.1	40.2 - 47.9
	2005	1,308,000	73.0	68.5 - 77.6

### Surveillance Data and Method for Estimating Incidence

The data of sentinels' report of influenza and pediatric diseases from week 1 of 2002 through week 52 of 2005 in the NESID in Japan were used. The numbers of all medical institutions were obtained from the National Survey of Medical Care Institutions conducted by the Ministry of Health, Labour and Welfare in October 2002.^17^

The annual and weekly incidence was estimated using the method proposed by Hashimoto et al.^12^ For each disease, prefecture and type of medical institution, the incidences in sentinels follow a multi-hypergeometric distribution under the fixed condition of the total number of sentinels under the assumption that sentinels are randomly selected from all medical institutions. The total incidence in each prefecture and type of medical institution were estimated as the total incidence in sentinels divided by the proportion of sentinels to all medical institutions. The total incidence in all medical institutions was estimated to be the total of those in all prefectures and types of medical institution. The approximate confidence interval for the incidence was given based on the distribution. The appendix shows the method for estimating incidences in detail.

Types of medical institutions were classified using the information from the National Survey of Medical Care Institutions as follows; three types for pediatric diseases: "pediatric department in hospital," "clinic with pediatric department as its main department" and "clinic with pediatric department not as its main department." For influenza, the three types above were used plus "department of internal medicine in hospital, and clinic with internal medicine but without pediatric department."

Table 1 shows the numbers of all and sentinel medical institutions by type of medical institution. The number of sentinels in 2002-2005 was about 4,700 for influenza and 3,100 for pediatric diseases. The proportion of sentinels in all medical institutions was 7.1% for influenza and 11.5-11.6% for pediatric diseases.

**Table 1.** The number of all and sentinel medical institutions by type of medical institution, Japan, 2002-2005.

	No. of all medical institutions	No. of sentinel medical institutions (%)			

		2002	2003	2004	2005
Influenza					
Total	66,014	4,659 (7.1)	4,672 (7.1)	4,679 (7.1)	4,693 (7.1)
Pediatric department in hospital	2,859	643 (22.5)	656 (22.9)	597 (20.9)	592 (20.7)
Clinic with pediatric department as its main department	5,483	1,816 (33.1)	1,831 (33.4)	1,838 (33.5)	1,844 (33.6)
Clinic with pediatric department not as its main department	18,156	1,093 (6.0)	1,108 (6.1)	1,103 (6.1)	1,093 (6.0)
Department of internal medicine in hospital, and clinic with internal medicine but without pediatric department	39,516	1,107 (2.8)	1,077 (2.7)	1,141 (2.9)	1,164 (2.9)
Pediatric diseases					
Total	26,498	3,057 (11.5)	3,077 (11.6)	3,062 (11.6)	3,086 (11.6)
Pediatric department in hospital	2,859	737 (25.8)	734 (25.7)	733 (25.6)	732 (25.6)
Clinic with pediatric department as its main department	5,483	1,779 (32.4)	1,804 (32.9)	1,806 (32.9)	1,810 (33.0)
Clinic with pediatric department not as its main department	18,156	541 (3.0)	539 (3.0)	523 (2.9)	544 (3.0)

The number of all medical institutions was obtained from the National Survey of Medical Care Institutions in 2002. Proportion of sentinel medical institutions in all medical institutions in parentheses.

### Method of Analysis

Incidence rate per population was calculated using the incidence estimated above and the 2003 population in Japan. For influenza, the sex- and age-specific annual and weekly incidence rates were calculated. The proportion of weekly incidence to each influenza season's total incidence was presented by age group. Age groups were the following three; 0-14, 15-59, and 60 years old or over. In pediatric diseases, annual and weekly incidence rates were calculated for population aged 0-14 years.

## RESULTS

### Influenza

Table 2 shows the annual incidence rates of influenza by sex and age. The annual incidence rate per 1,000 population was 57.7 (95% confidence interval [CI]: 54.5-60.7) in 2002, 90.6 (95% CI: 86.7-94.4) in 2003, 70.1 (95% CI: 67.2-73.1) in 2004, and 142.6 (95% CI: 135.6-149.6) in 2005. The difference in incidence rates between male and female was not so large. The incidence rate in the 0-14 years age group was higher than in other age groups.

**Table 2.** Estimated incidence rates of influenza by sex and age, Japan, 2002-2005.

Year	Sex	Age (years)	Estimated incidence	Incidence rate (per 1,000 population)		

				Estimate	95% confidence interval	
2002	Total	Total	7,360,000	57.7	54.5	- 60.7

	Male	Total	3,740,000	60.0	56.8	- 63.2
		0-14	2,190,000	238.7	221.2	- 256.1
		15-60	1,420,000	36.6	34.8	- 38.4
		60 and over	130,000	9.1	8.4	- 9.8

	Female	Total	3,620,000	55.4	52.4	- 58.5
		0-14	1,950,000	223.4	206.2	- 239.4
		15-60	1,510,000	39.5	37.6	- 41.6
		60 and over	160,000	8.7	7.6	- 9.3

2003	Total	Total	11,560,000	90.6	86.7	- 94.4

	Male	Total	5,800,000	93.1	89.1	- 97.1
		0-14	3,160,000	344.4	324.8	- 364.0
		15-60	2,330,000	60.0	56.9	- 63.1
		60 and over	310,000	21.7	21.0	- 23.1

	Female	Total	5,760,000	88.2	84.5	- 91.9
		0-14	2,820,000	323.1	304.7	- 341.4
		15-60	2,560,000	66.9	63.8	- 70.0
		60 and over	380,000	20.7	19.6	- 21.8

2004	Total	Total	8,950,000	70.1	67.2	- 73.1

	Male	Total	4,500,000	72.2	69.2	- 75.4
		0-14	2,220,000	241.9	229.9	- 252.8
		15-60	2,040,000	52.5	49.7	- 55.3
		60 and over	250,000	17.5	16.1	- 18.9

	Female	Total	4,450,000	68.1	65.2	- 70.9
		0-14	1,970,000	225.7	214.2	- 237.1
		15-60	2,160,000	56.5	53.8	- 59.3
		60 and over	310,000	16.9	15.8	- 18.0

2005	Total	Total	18,200,000	142.6	135.6	- 149.6

	Male	Total	9,020,000	144.8	137.2	- 152.2
		0-14	4,500,000	490.4	465.3	- 516.6
		15-60	3,790,000	97.6	90.1	- 105.0
		60 and over	730,000	51.1	46.2	- 56.0

	Female	Total	9,180,000	140.5	134.0	- 147.1
		0-14	4,030,000	461.7	437.6	- 484.6
		15-60	4,260,000	111.3	104.6	- 117.9
		60 and over	890,000	48.6	44.2	- 52.9

**Figure 1.**
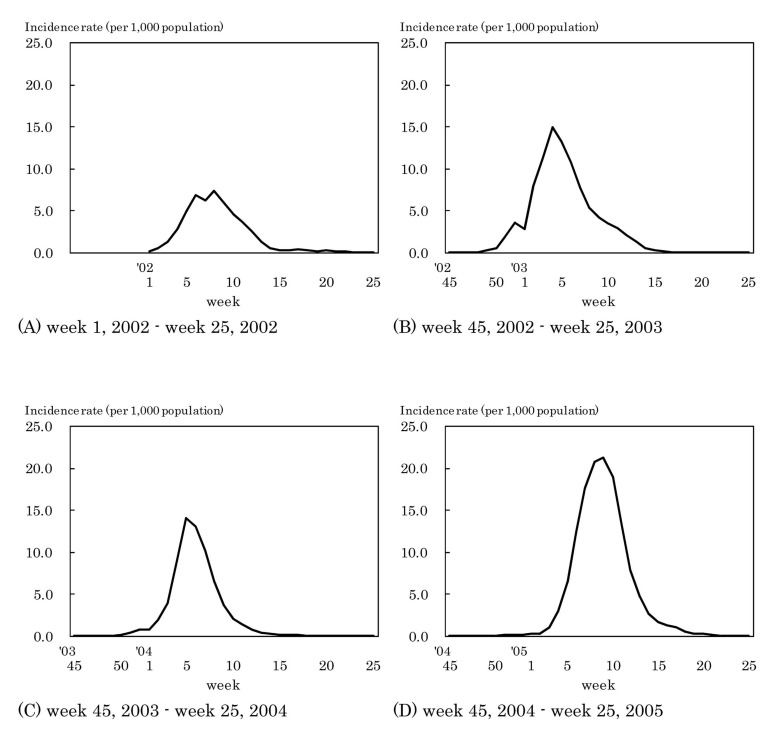
Estimated incidence rates of influenza by week, Japan, 2002-2005.

Figure 1 shows the weekly incidence rates of influenza. The highest weekly incidence rate per 1,000 population was 7.4 in week 8 of 2002, 14.9 in week 4 of 2003, 14.1 in week 5 of 2004, and 21.2 in week 9 of 2005. The period with an incidence rate of 1.0 or more was as follows: from week 3 to week 13 of 2002, from week 51 of 2002 to week 13 of 2003, from week 2 to week 11 of 2004, and from week 3 to week 17 of 2005.

**Figure 2.**
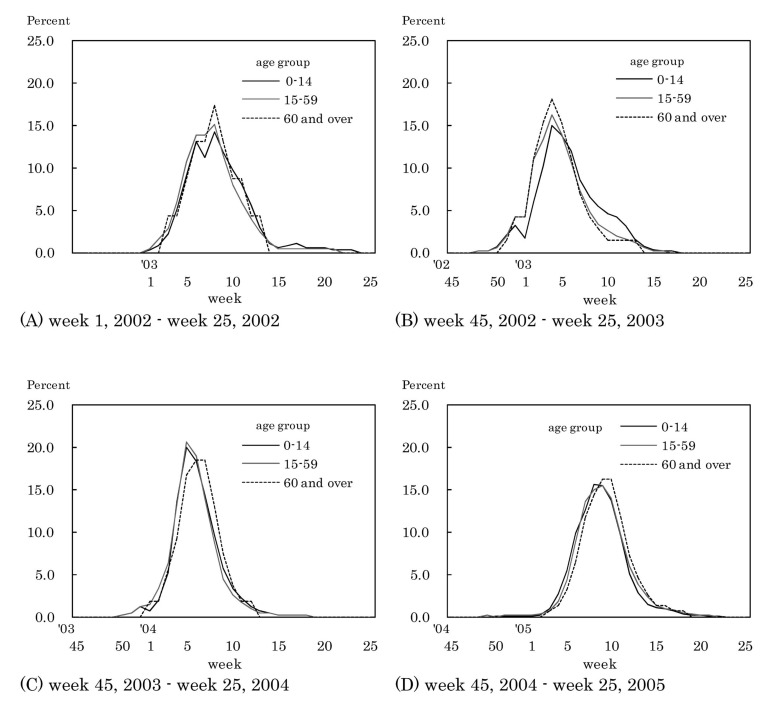
Proportion of weekly incidence in relation to each influenza season's total incidence by age group, Japan, 2002-2005.

Figure 2 shows the proportion of weekly incidence in relation to each influenza season's total incidence by age. The peak week in the proportions in every age group was week 8 of 2002 in the 2001/2002 season and week 4 of 2003 in the 2002/2003 season. In the 2003/2004 season, the peak week was week 5 of 2004 in the aged 0-14 and 15-59 groups, and week 6-7 of 2004 in those aged 60 and over. In the 2004/2005 season, the peak week was week 8 in those aged 0-14, week 9 in those aged 15-59 and week 9-10 in those aged 60 and over.

### Pediatric Diseases

Table 3 shows the incidence rates of pediatric diseases per 1,000 population among persons aged 0-14 years. The incidence rate in 2002-2005 was less than 5.0 for pertussis, rubella, and measles, 293.2-320.8 for infectious gastroenteritis, and 5.3-89.6 for other 8 diseases.

**Figure 3.**
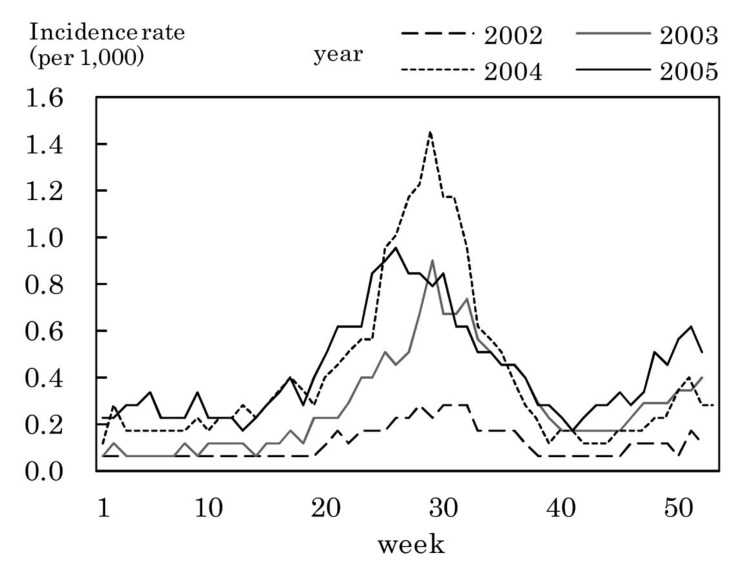
Estimated incidence rates of pharygoconjunctival fever by week, Japan, 2002-2005.

**Figure 4.**
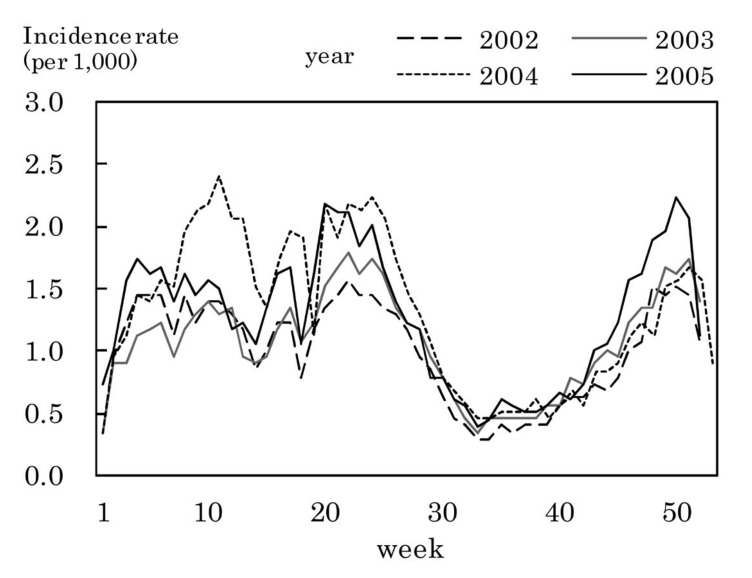
Estimated incidence rates of group A streptococcal pharyngitis by week, Japan, 2002-2005.

**Figure 5.**
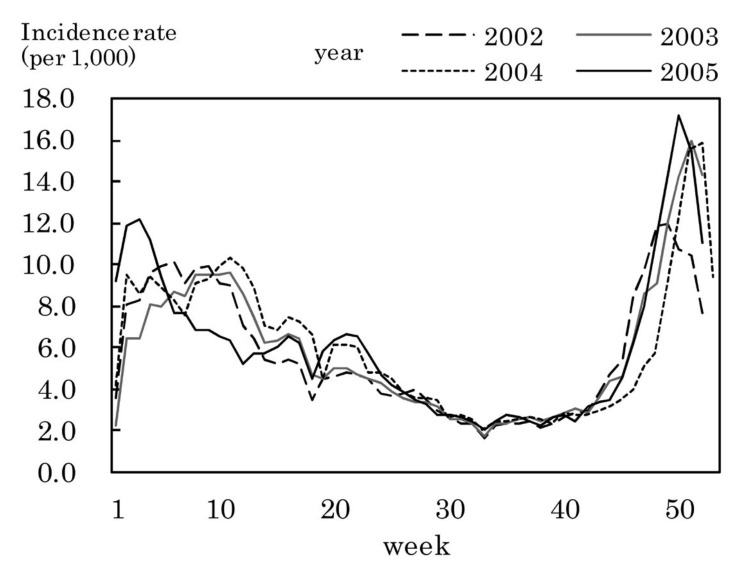
Estimated incidence rates of infectious gastroenteritis by week, Japan, 2002-2005.

**Figure 6.**
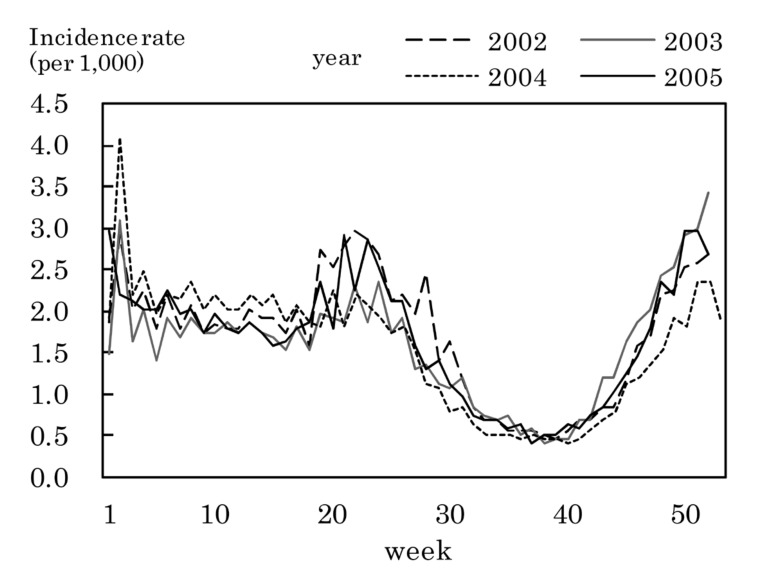
Estimated incidence rates of chickenpox by week, Japan, 2002-2005.

**Figure 7.**
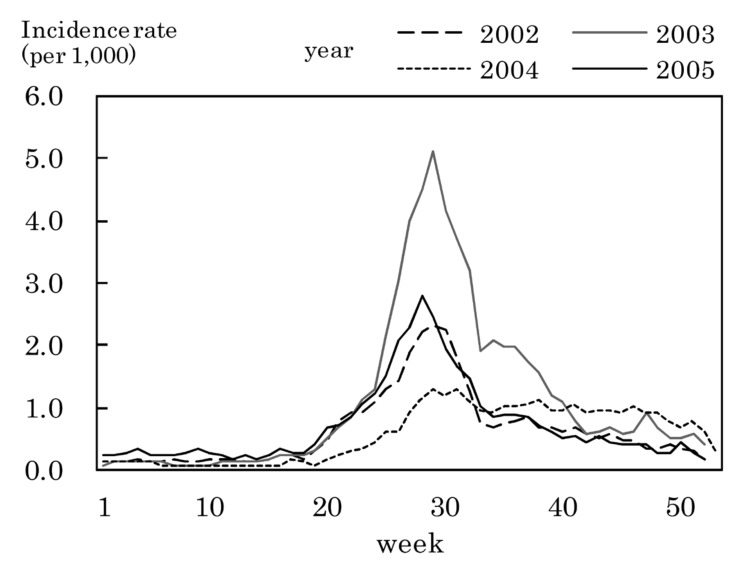
Estimated incidence rates of hand-foot-mouth disease by week, Japan, 2002-2005.

**Figure 8.**
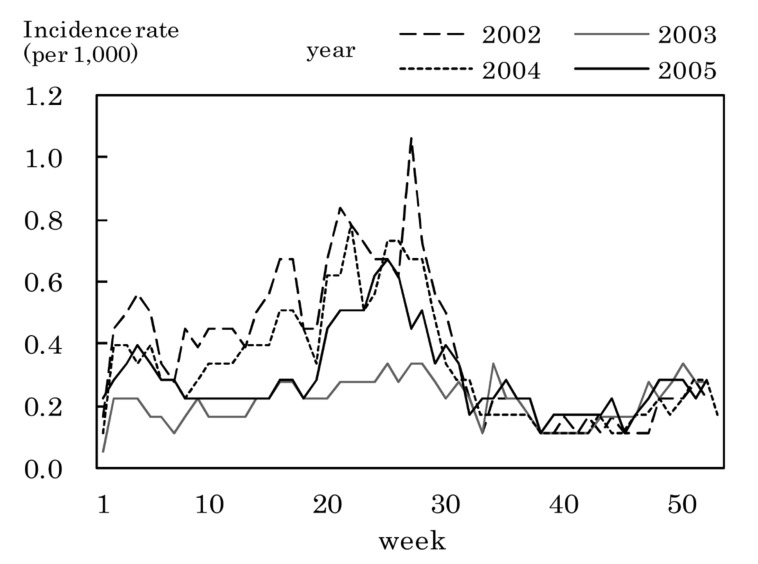
Estimated incidence rates of erythema infectiosum by week, Japan, 2002-2005.

**Figure 9.**
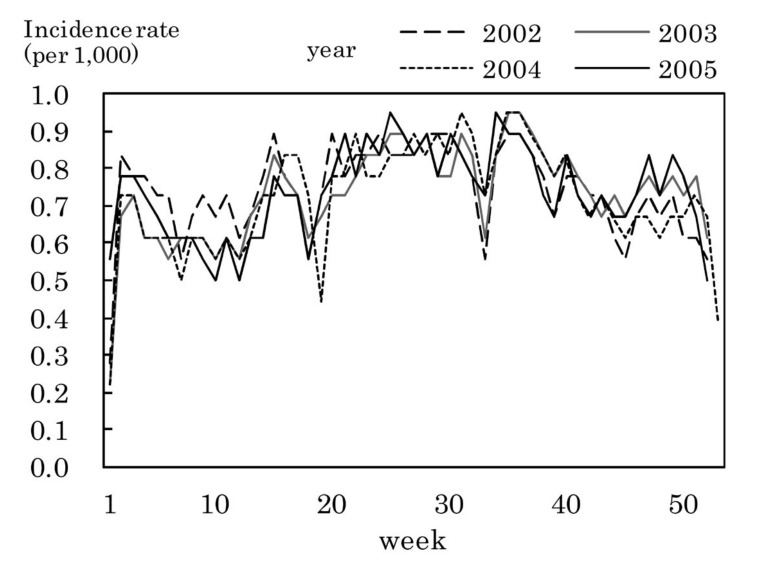
Estimated incidence rates of exanthem subitum by week, Japan, 2002-2005.

**Figure 10.**
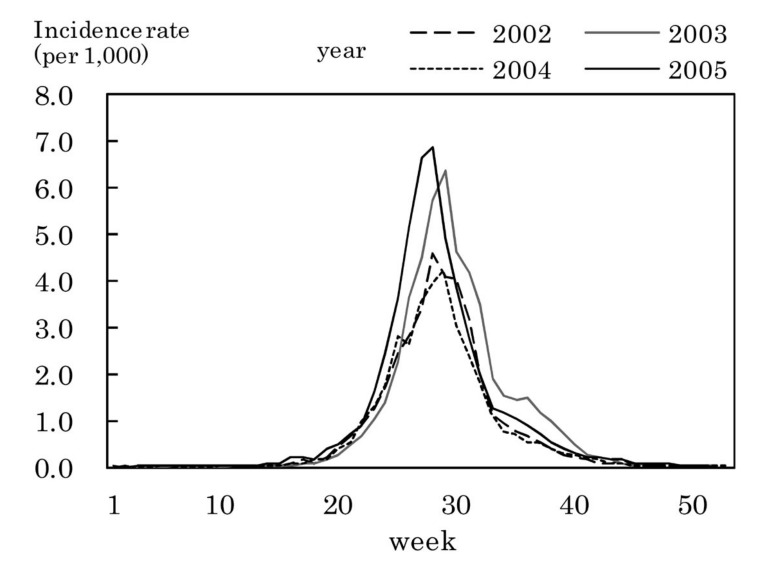
Estimated incidence rates of herpangina by week, Japan, 2002-2005.

**Figure 11.**
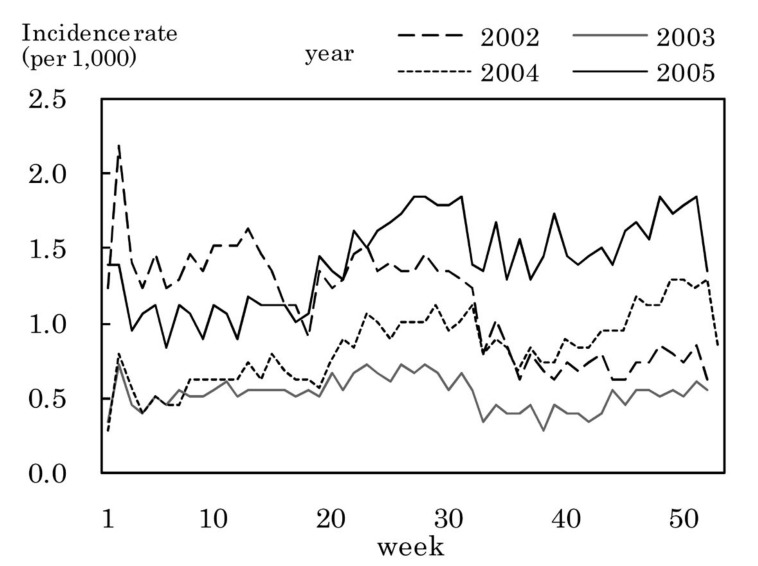
Estimated incidence rates of mumps by week, Japan, 2002-2005.

Figures 3 to 11 shows the weekly incidence rates of 9 pediatric diseases per 1,000 population among those 0-14 years old, respectively. For pertussis, rubella, and measles, they were not shown because of their low annual incidence rates. The seasonal pattern was observed each year in many diseases. The highest weekly incidence rate in the four years was less than 1.0 for exanthem subitum (Figure 9), more than 5.0 for infectious gastroenteritis (Figure 5), hand-foot-mouth disease (Figure 7) and herpangina (Figure 10), and 1.0-5.0 in the other five diseases.

## DISCUSSION

Large yearly and seasonal variation, small sex difference and age distribution with higher incidence rate in younger population were observed in the incidence rates of influenza. These results were similar to those in previous studies.^1^^-^^3^ The highest weekly incidence rate in 2002-2005 was 7.4-21.2 per 1,000 population. This would provide useful information for preventive countermeasures against the epidemic spread of influenza. The week with the highest weekly incidence rate in the over-sixties bracket was later than that in the 0-14 years of age bracket in 2004 and 2005, while such a phenomenon was not observed in 2002 and 2003. This finding would be related to several factors such as combination of epidemics of different virus types, its difference between years, proportion of persons with susceptibility to the virus, its difference between younger and older population.^18^

The incidence rate in the population aged 0-14 years in 2002-2005 was low in pertussis, rubella, and measles, presumably due to the association with the vaccination program against these diseases in Japan.^19^^,^^20^ Some seasonal patterns were observed in many pediatric diseases as shown in Figures 3 to 11. These results were obtained in other previous reports.^4^^-^^6^ The highest weekly incidence rate per 1,000 population aged 0-14 years in 2002-2005 was less than 1.0 for exanthem subitum. It is related to little seasonal variation. The rate was more than 5.0 for infectious gastroenteritis, hand-foot-mouth disease and herpangina. This is related to the high incidence rate of infectious gastroenteritis, and the large seasonal variation in hand-foot-mouth disease and herpangina. This finding means that the epidemic of these three diseases spread rapidly, and would be important for planning control of their epidemics.

There are some limitations and problems in the present study. The main problems would be in the data and method for estimating the incidence. Problems with the data in the reports to the NESID in Japan include the inaccuracy of disease diagnosis and incompleteness of reporting.^13^ Those in the method have been already discussed in the previous reports in detail.^12^^,^^16^ The assumption in the method that sentinels are randomly selected from all medical institutions is critical. Although the NESID guidelines in Japan calls for the sentinels to be selected from all medical institutions in public health areas as randomly and as representatively as possible, sentinels seem to be recruited on a voluntary basis to some extent. It was reported that the mean size of the underlying population was larger in sentinels than in all medical institutions, that the incidence was overestimated because the assumption was failing, and that the ratio of the estimated to the actual incidence of influenza and pediatric diseases based on the sentinel surveillance data in the NESID in Japan would be 1.06-1.26.^12^

In conclusion, we estimated the annual and weekly incidence rates of influenza and pediatric diseases in Japan in 2002-2005, and described their temporal variation.

## APPENDIX

Consider the distribution of incidences in medical institutions. Let *m* be an integer greater than the largest incidence among medical institutions, *n* be the number of all medical institutions, and *n_i_* be the number of medical institutions with the incidence of *i* for *i* = 0, 1, ···, *m*. Let *N* be the number of sentinels, and *N_i_* be the number of sentinels with the incidence of *i* for *i* = 0, 1, ···, *m*. The constants of *n* and *N* are known, and those of {*n_i_*} are unknown. {*N_i_*} are obtained from the sentinel surveillance, and follow a multihypergeometric distribution under the condition of *N* fixed under the assumption that sentinels are randomly selected in all medical institutions.

Let α be the total incidence in all medical institutions, and note that α = Σ*i* × *n_i_*. The estimate of α is given to be α = Σ*i* × *N_i_* × *n*/*N*, i.e., the incidence is estimated as the total incidence in sentinels (Σ*i* × *N_i_*) divided by the proportion of sentinels among all medical institutions (*N*/*n*).

The approximate confidence interval for α is given to be $\left(\hat{\alpha }-1.96\times s,\;\hat{\alpha }+1.96\times s\right)$, where *s*^2^ is an estimate of variance of α and is given to be:

[Σ*i*^2^ × *N_i_*/*N* − (Σ*i* × *N_i_*/*N*)^2^] × *n*^3^ (1/*N* − 1/*n*)/(*n* − 1).

Consider that the incidences in some strata such as prefectures and types of medical institution are estimated using the above-explained method. Let *k* be the number of strata, ${\hat{\alpha }}_{1},{\hat{\alpha }}_{2},\cdots ,\alpha_{k}$, the estimated incidences in the strata, and $s_{1}^{2},s_{2}^{2},\cdots ,s_{k}^{2}$, their estimated variances. The approximate confidence interval for the total incidence is given as $\left({\hat{\alpha }}_{t}-1.96\times s_{t},{\hat{\alpha }}_{t}+1.96\times s_{t}\right)$, where ${\hat{\alpha }}_{t}={\hat{\alpha }}_{1}+,{\hat{\alpha }}_{2}+\cdots +\;\alpha_{k}$ and $s_{t}^{2}=s_{1}^{2}+s_{2}^{2}+\cdots +s_{k}^{2}$.
